# Genetically Engineered Cellular Nanovesicle as Targeted DNase I Delivery System for the Clearance of Neutrophil Extracellular Traps in Acute Lung Injury

**DOI:** 10.1002/advs.202303053

**Published:** 2023-09-27

**Authors:** Yang Du, Yining Chen, Fangyuan Li, Zhengwei Mao, Yuan Ding, Weilin Wang

**Affiliations:** ^1^ Department of Hepatobiliary and Pancreatic Surgery The Second Affiliated Hospital Zhejiang University School of Medicine Hangzhou Zhejiang 310009 China; ^2^ Key Laboratory of Precision Diagnosis and Treatment for Hepatobiliary and Pancreatic Tumor of Zhejiang Province Hangzhou Zhejiang 310009 China; ^3^ Research Center of Diagnosis and Treatment Technology for Hepatocellular Carcinoma of Zhejiang Province Hangzhou Zhejiang 310009 China; ^4^ National Innovation Center for Fundamental Research on Cancer Medicine Hangzhou Zhejiang 310009 China; ^5^ Cancer Center Zhejiang University Hangzhou Zhejiang 310058 China; ^6^ ZJU‐Pujian Research & Development Center of Medical Artificial Intelligence for Hepatobiliary and Pancreatic Disease Hangzhou Zhejiang 310058 China; ^7^ Institute of Pharmaceutics Hangzhou Institute of Innovative Medicine College of Pharmaceutical Sciences Zhejiang University Hangzhou Zhejiang 310058 China; ^8^ MOE Key Laboratory of Macromolecular Synthesis and Functionalization Department of Polymer Science and Engineering Zhejiang University Hangzhou Zhejiang 310027 China

**Keywords:** acute lung injury, biomimetic nanovesicles, bioorthogonal chemistry, genetic engineering, neutrophil extracellular traps, protein delivery

## Abstract

Acute lung injury (ALI)/acute respiratory distress syndrome (ARDS) are prevalent critical illnesses with a high mortality rate among patients in intensive care units. Neutrophil extracellular traps (NETs) are implicated in the pathogenesis of ALI/ARDS and represent a promising therapeutic target. However, the clinical application of deoxyribonuclease I (DNase I), the only drug currently available to clear NETs, is limited due to the lack of precise and efficient delivery strategies. Therefore, targeted delivery of DNase I to the inflamed lung remains a critical issue to be addressed. Herein, a novel biomimetic DNase I delivery system is developed (DCNV) that employs genetically and bioorthogonally engineered cellular nanovesicles for pulmonary NETs clearance. The CXC motif chemokine receptor 2 overexpressed cellular nanovesicles can mimic the inflammatory chemotaxis of neutrophils in ALI/ARDS, leading to enhanced lung accumulation. Furthermore, DNase I immobilized through bioorthogonal chemistry exhibits remarkable enzymatic activity in NETs degradation, thus restraining inflammation and safeguarding lung tissue in the lipopolysaccharide‐induced ALI murine model. Collectively, the findings present a groundbreaking proof‐of‐concept in the utilization of biomimetic cellular nanovesicles to deliver DNase I for treating ALI/ARDS. This innovative strategy may usher in a new era in the development of pharmacological interventions for various inflammation‐related diseases.

## Graphical abstract

A biomimetic deoxyribonuclease I (DNase I) delivery system (DCNV) that employs CXC motif chemokine receptor 2 overexpressed cellular nanovesicles for pulmonary NETs clearance is reported. DNase I is immobilized on the surface of DCNV through biorthogonal chemistry with high enzymatic activity preserved. DCNV mimics the chemotaxis of neutrophils during inflammation to improve the accumulation of DNase I in the inflamed lung, thereby alleviating acute lung injury.

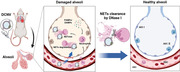

## 1 Introduction

Acute lung injury (ALI) / acute respiratory distress syndrome (ARDS) are the pulmonary manifestations of an inflammatory condition characterized by non‐cardiogenic pulmonary edema and hypoxemia.^[^ ^1^ ^]^ ALI/ARDS remains a life‐threatening disease in intensive care unit patients, with a high mortality rate of up to 45%.^[^ ^2^ ^]^ Mechanical ventilation and fluid management are the current treatment options for ALI/ARDS, but they are only capable of providing symptomatic relief.^[^ ^3^ ^]^ Moreover, the efficacy of existing drugs for treating ALI/ARDS is controversial.^[^ ^4^ ^]^ For instance, the clinical application of glucocorticoids is limited by their non‐specific immunosuppressive effects and associated adverse reactions, which may cause harm in certain subgroups of patients and do not improve longer‐term outcomes.^[^ ^5^ ^]^ Therefore, the development of precise and effective pharmacological interventions for ALI/ARDS is urgently needed.

Neutrophil infiltration and release of neutrophil extracellular traps (NETs) have recently been identified as crucial pathological processes in the development of ALI/ARDS.^[^ ^6^ ^]^ Notably, the CXC motif chemokine receptor, especially CXCR2, is capable of recognizing elevated CXC chemokine ligands (CXCLs) within inflamed lungs, mediating the recruitment and migration of neutrophils during ALI/ARDS.^[^ ^7^ ^]^ Once neutrophils are recruited, they become activated in response to pro‐inflammatory cytokines and damage‐associated molecular patterns (DMAPs), triggering the release of NETs, a fibrous structure composed of decondensed chromatin and granular components.^[^ ^6a^ ^]^ The formation of NETs further exacerbates lung tissue damage and promotes inflammation in different types of ALI/ARDS.^[^ ^8^ ^]^ Recent research on COVID‐19 ARDS patients has also demonstrated that elevated levels of NETs in the bloodstream are closely associated with poor patient outcomes.^[^ ^9^ ^]^ Thus, effectively clearing NETs from inflammatory lung tissues may be a potential therapeutic strategy for treating ALI/ARDS.

Deoxyribonuclease I (DNase I), an enzyme that can break down the nucleic acid backbone of NETs is a promising drug candidate for their clearance.^[^ ^10^ ^]^ DNase I inhalation therapy has been approved by the US Food and Drug Administration in cystic fibrosis.^[^ ^11^ ^]^ However, the current inhalation delivery is not suitable for ARDS patients, who typically require mechanical ventilation support. Administering inhalation therapy during mechanical ventilation presents several limitations, including low drug delivery efficiency and the risk of aerosol transmission in patients with infectious ARDS.^[^ ^12^ ^]^ Furthermore, the short half‐life and high clearance rate of DNase I limit its intravenous administration.^[^ ^13^ ^]^ Although previous studies indicated that loading DNase I into liposomes or polymers can prolong its half‐life in blood plasma, these carriers lack active targeting capabilities to facilitate the effective accumulation of DNase I in inflamed lung tissues.^[^ ^14^ ^]^ In addition, direct encapsulation of DNase I into liposomes may hinder its catalytic efficiency, as the lipid bilayers act as a barrier that impedes the interaction between DNase I and its substrates.^[^ ^14a^ ^]^ Therefore, achieving targeted delivery of DNase I to the inflammatory sites of ALI/ARDS and ensuring its efficient catalysis remains a great challenge that needs to be addressed.

As nanobioengineering technology continues to advance, biomimetic nano‐delivery systems with superior disease‐targeting capabilities and biocompatibility are gaining ever‐growing interest in the biomedical field. Biomimetic nanovesicles are a type of nano‐carrier prepared from natural or gene‐engineered biological membranes, which possess relevant membrane proteins and a phospholipid bilayer structure.^[^ ^15^ ^]^ They can utilize the adhesive molecules and chemokine receptors expressed on their surface to specifically target inflammatory environments, improving the delivery efficiency of loaded drugs.^[^ ^16^ ^]^ Meanwhile, these biological membranes can be further functionalized by chemical modifications.^[^ ^17^ ^]^ Bioorthogonal chemistry is one of the most powerful methods for attaching functional components to biological membranes through intrinsic cellular metabolic processes, without compromising the function of membrane proteins.^[^ ^18^ ^]^ Recently, this technology has been applied to the decoration of living cells or cellular nanovesicles with functional proteins, including hyaluronidase and T cell stimulants, thereby endowing them with capabilities such as tissue penetration and immune regulation.^[^ ^19^ ^]^ Conjugating DNase I onto the surface of biomimetic nanovesicles via bioorthogonal chemistry has the potential to overcome the barrier effect of the phospholipid bilayer, leading to enhanced interactions between DNase I and NETs. Additionally, the mild reaction conditions used in this approach could also preserve the structural and functional integrity of both membrane proteins and DNase I, making it a promising strategy for fabricating DNase I delivery systems that can efficiently target inflammation and degrade NETs.

**Figure 1:**
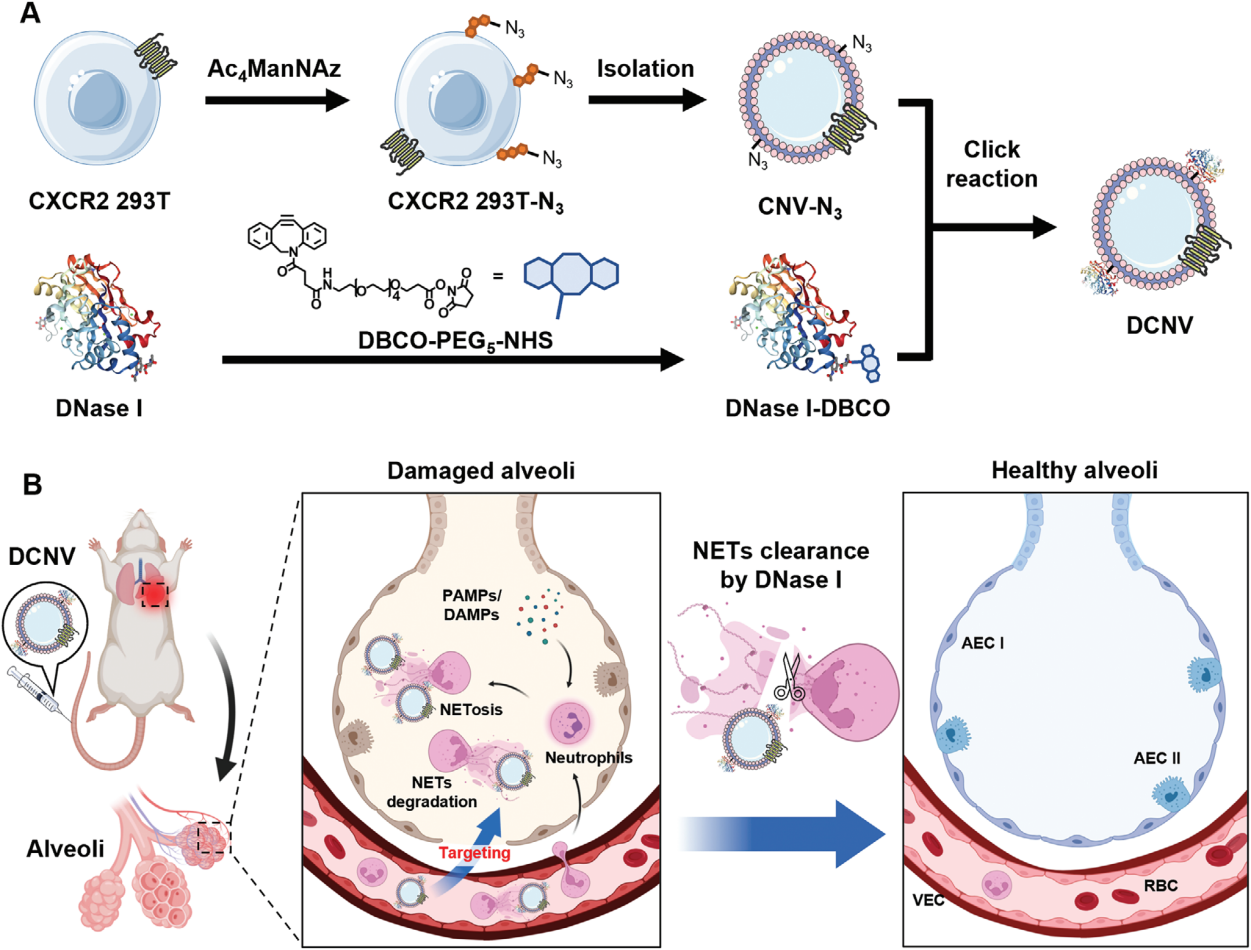
Schematic illustration of the preparation of DCNV and the proposed strategy for treating ALI/ARDS. A) CXCR2 293T cells are cultured with Ac_4_ManNAz to prepare azido‐functionalized biomimetic nanovesicles (CNV‐N_3_). Subsequently, DBCO‐modified DNase I is immobilized on the surface of CNV‐N_3_ through click chemistry to obtain DCNV. B) After intravenous injection, DCNV can effectively target the inflamed lung with the help of surface‐expressed CXCR2 and degrade NETs through surface‐decorated DNase I, thereby alleviating lung inflammation in ALI/ARDS.

In this work, we developed a biomimetic DNase I delivery system (DCNV) using genetically and bioorthogonally engineered cellular nanovesicles for targeted NETs clearance in ALI/ARDS. Given the previous findings indicating the CXCR2‐dependent recruitment of neutrophils in ARDS, we engineered HEK 293T cells with CXCR2 overexpression (CXCR2 293T) to mimic this chemotactic process.^[^ ^7^, ^20^ ^]^ Then, we prepared azido‐functionalized biomimetic nanovesicles (CNV‐N_3_) from these cells through Ac_4_ManNAz‐mediated metabolic engineering. Finally, dibenzocyclooctyne (DBCO)‐modified DNase I (DNase I‐DBCO) was conjugated onto the surface of CNV‐N_3_ to obtain DCNV. We demonstrated that upon intravenous injection, DCNV can effectively accumulate in inflamed lungs to degrade NETs and reduce lung inflammation in the lipopolysaccharide (LPS)‐induced ALI mouse model (**Figure** **1**). Our study provides the first proof‐of‐concept for the delivery of DNase I using biomimetic cellular nanovesicles, which presents a promising therapeutic intervention strategy for ALI/ARDS.

## 2 Results and Discussion

### 2.1 Preparation and Characterization of DCNV

**Figure 2:**
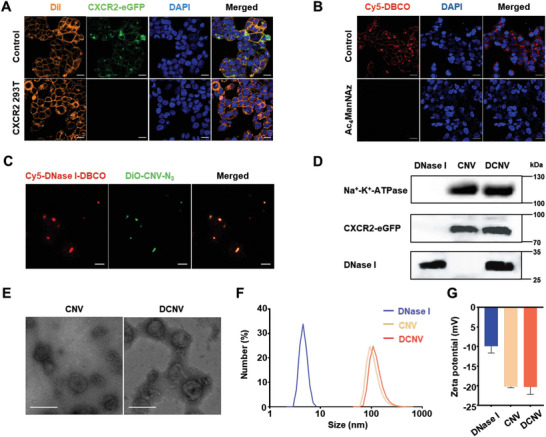
Preparation and Characterization of DCNV. A) Confocal laser scanning microscope (CLSM) images of wild‐type 293T cells and CXCR2 293T cells. DiI‐stained cell membrane (orange), CXCR2‐eGFP (green), and DAPI‐labeled nucleus (blue). Scale bar = 20 µm. B) CLSM images of CXCR2 293T cells treated with or without Ac_4_ManNAz and then incubated with Cy5‐DBCO. Cy5‐DBCO (red) and DAPI‐labeled nucleus (blue). Scale bar = 20 µm. C) CLSM images of Cy5‐NHS‐labeled DNase I‐DBCO and DiO‐labeled CNV‐N_3_. Cy5‐labeled DNase I‐DBCO (red) and DiO‐labeled CNV‐N_3_ (green). Scale bar = 10 µm. D) Western blot analysis of DCNV. E) TEM images of CNV and DCNV. Scale bar = 200 nm. F) Hydrodynamic size distribution of DNase I, CNV, and DCNV in PBS. G) Zeta potential of DNase I, CNV, and DCNV in PBS (n = 3).

The preparation of DCNVs involves five steps: 1) construction of a CXCR2 overexpressed cell line, 2) surface‐functionalization of the cell with azide groups, 3) generation of CNV‐N_3_, 4) conjugation of DNase I with the DBCO moiety, and 5) anchoring the DNase I‐DBCO to CNV‐N_3_. First, HEK 293T cells were selected for constructing the CXCR2 overexpressed cell line due to their rapid mitotic cycle and remarkable transfection capabilities to yield abundant recombinant proteins.^[^ ^21^ ^]^ To visualize the expression of CXCR2, an eGFP protein tag was added to the C‐terminal portion of CXCR2, positioned close to the inner leaflet of cell membranes, while the functional domain of CXCR2 was located extracellularly. As shown in **Figure** **2**A, CXCR2‐eGFP protein (green) was strongly expressed in CXCR2 transfected cells and colocalized with the cell membrane stained by DiI (yellow), confirming the successful transfection. Furthermore, the expression of CXCR2 on the surface of CXCR2 293T cells was further validated by flow cytometry using anti‐CXCR2 antibodies (Figure S1, Supporting Information). After that, CXCR2 293T cells were incubated with Ac_4_ManNAz, which facilitated the integration of azide groups on their surface through glucose metabolism. The successful functionalization of azide groups on the surface of CXCR2 293T cells was confirmed by red fluorescence upon treatment with cycloalkyne dyes (Cy5‐DBCO) (Figure 2B). The cytoplasmic staining of azide groups may be attributed to the dynamic recycling of the azide‐decorated vesicles, shuttling between the cell membrane and various subcellular organelles for metabolic processes.^[^ ^22^ ^]^ Then, cell membranes from azide‐functionalized CXCR2 293T were collected by repeated freeze‐thaw process and extruded through filters with progressively diminishing pore sizes to form CNV‐N_3_.^[^ ^23^ ^]^ Meanwhile, DBCO‐PEG_5_‐NHS was employed to prepare DBCO‐functionalized DNase I via an amine─NHS coupling reaction. The detection of a distinct absorption peak at 308 nm by UV–vis spectroscopy provided evidence for the successful conjugation of the DBCO group onto DNase I. The degree of functionalization was quantified to be 1.05 DBCO moieties per molecule of DNase I (Figure S2, Supporting Information). Finally, DCNV was obtained by decorating CNV‐N_3_ with DNase I‐DBCO using copper‐free click chemistry. To visualize the connections between DNase I‐DBCO and CNV‐N_3_, Cy5‐NHS and DiO were utilized to label DNase I and CNV, respectively. The colocalization of red and green fluorescence from Cy5‐labeled DNase I (Cy5‐DNase I‐DBCO) and DiO‐labeled CNV‐N_3_ (DiO‐CNV‐N_3_) suggested the successful immobilization of DNase I (Figure 2C). In addition, the successful preparation of DCNV was confirmed by Western blot analysis, which revealed distinct patterns of CXCR2‐eGFP and DNase I (Figure 2D). By controlling the feed ratio, the grafting efficiency of DNase I can be modulated, with an optimal value of ≈27.7 µg DNase I for every 100 µg DCNV achieved at a 1:1 feed ratio of DNase I to CNV‐N_3_ (Figure S3, Supporting Information). Transmission electron microscopy (TEM) analysis revealed that both CNV and DCNV displayed spherical nanostructures (Figure 2E). The hydrodynamic diameter of DCNV was measured to be 108 nm according to dynamic light scattering (DLS) analysis. Notably, the hydrodynamic size of DCNV increased by ≈16 nm after decoration with DNase I (Figure 2F). Moreover, DCNV exhibited a zeta potential of −20.3 mV (Figure 2G), which is consistent with the negative surface charge of biological membranes.^[^ ^19b^ ^]^

### 2.2 DCNV Preserved the Enzymatic Activity of DNase I and Degraded NETs in Vitro

**Figure 3:**
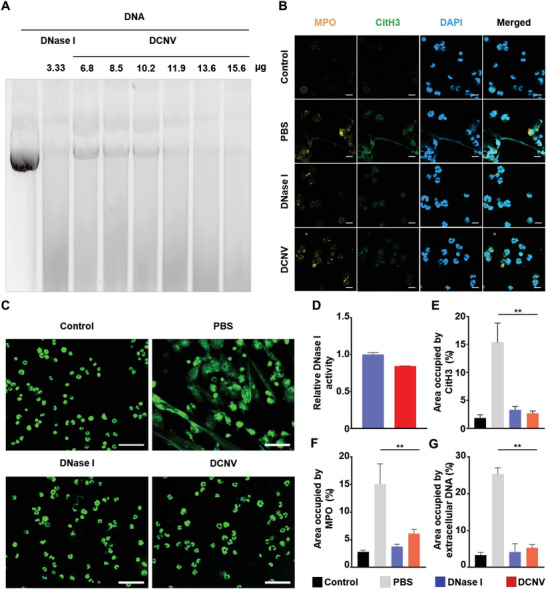
In vitro NETs degradation activity of DCNV. A) DNA digestion and electrophoresis assay of DNase I and DCNV. B) Representative CLSM images of NETs immunostaining from the following groups: neutrophils without PMA induction (Control), PMA‐treated neutrophils with PBS treatment (PBS), PMA‐treated neutrophils with DCNV treatment (DCNV), and PMA‐treated neutrophils with DNase I treatment (DNase I). CitH3 (green), MPO (yellow), and DAPI (blue). Scale bars = 10 µm. C) Representative CLSM images of SYTOX green‐stained NETs. Scale bar = 10 µm. D) Relative enzymatic activity of DCNV (n = 3). E) NETs quantification by the area occupied by CitH3 (n = 5). F) NETs quantification by the area occupied by MPO (n = 5). G) NETs quantification by the area occupied by extracellular DNA (n = 5). ^**^p < 0.01.

Efficient clearance of NETs requires the immobilization of DNase I without compromising its enzymatic activity. Thus, the enzymatic activity of DCNV was assessed using pBR322 plasmid as the substrate, a 4362 bp double‐stranded DNA, through agarose gel electrophoresis.^[^ ^24^ ^]^ A total of 10 µg of pBR322 plasmid DNA could be fully degraded by either 3.33 µg (5 U) of free DNase I or DCNV over 13.6 µg, which is equivalent to 3.77 µg of immobilized DNase I (**Figure** **3**A). Moreover, the enzymatic activity of DCNV was further quantified by a DNase activity fluorometric assay kit. The results revealed that DNase I immobilized on DCNV retained ≈89% of the enzymatic activity of an equivalent amount of free DNase I (Figure 3D; Figure S4, Supporting Information). These findings suggested that DCNV preserves a high level of DNase I activity following immobilization via bioorthogonal chemistry.

To further confirm the NETs degradation capacity of DCNV, a NETs degradation assay was performed. The primary mouse neutrophils were first isolated from the tibia bone marrow, and their purity was determined to be 89.2% using flow cytometry (Figure S5, Supporting Information).^[^ ^25^ ^]^ Then, NETs were induced by treating isolated neutrophils with 100 nm phorbol 12‐myristate 13‐acetate (PMA).^[^ ^26^ ^]^ After 4 h of induction, free DNase I or DCNV (containing an equivalent amount of DNase I) was added to the culture media and incubated at 37 °C for 1 h. To evaluate NETs formation, markers such as extracellular myeloperoxidase (MPO) and citrullinated histone H3 (CitH3) were detected using immunofluorescence staining, while extracellular DNA of NETs was determined by SYTOX green staining.^[^ ^27^ ^]^ The strong colocalized fluorescence between MPO and CitH3 could be observed after PMA induction, indicating successful neutrophil activation and NETs formation. However, the fluorescence signal significantly decreased following treatment with DCNV, indicating the successful NETs degradation (Figure 3B). The extent of NETs degradation was quantified by measuring the CitH3‐stained area and MPO‐positive area (Figure 3E,F), which showed that DCNV exhibited comparable NETs degradation activity to that of free DNase I in vitro. Furthermore, SYTOX green staining produced consistent results with those described above (Figure 3C,G). All these results demonstrate that DCNV preserves the bioactivity of DNase I for NETs degradation in vitro.

### 2.3 DCNV Targeted to the Inflamed Lung in an ALI Mouse Model

**Figure 4:**
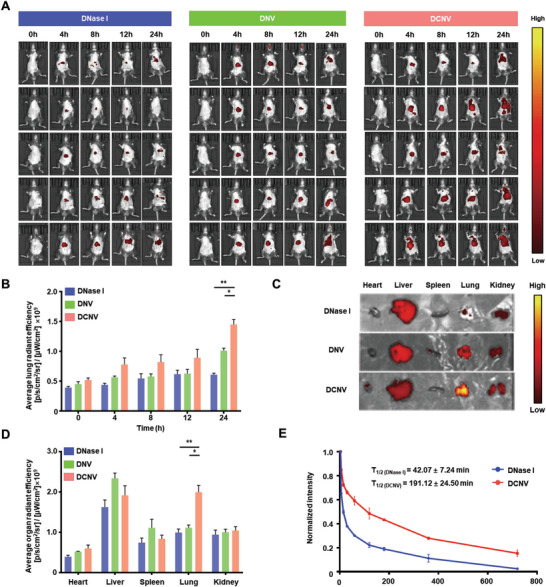
The lung‐targeting ability of DCNV in an ALI mouse model. A) IVIS images of ALI mice after intravenous injection with IR780‐labeled DNase I, DNV, and DCNV at indicated time points. B) Quantitative analysis of the fluorescence intensity in the lungs. C) Representative ex vivo fluorescence images of major organs (heart, liver, spleen, lung, and kidney) in different groups. D) Quantitative analysis of the fluorescence intensity in major organs (n = 5). E) Plasma pharmacokinetics of DNase I and DCNV following intravenous administration in ALI mice (n = 3). ^*^p <0.05; ^**^p <0.01.

The targeting ability of DCNV to the inflamed lung was further investigated in an LPS‐induced ALI mouse model. After 24 h of intratracheal LPS instillation, the mice were randomly divided into three groups and received intravenous injections of IR780‐labeled DNase I, DNV (DNase I decorated on the cellular nanovesicles without CXCR2 expression), and DCNV, respectively. Then, the biodistribution of DNase I, DNV, and DCNV was traced using an in vivo animal fluorescence imaging system (IVIS). As shown in **Figure** **4**A, the fluorescence signal of DNase I was mainly distributed in the liver and decreased at 12 h post‐injection, possibly due to its short half‐life and rapid elimination.^[^ ^13^ ^]^ In comparison to DNase I, DCNV demonstrated a noteworthy 4.54‐fold augmentation in blood half‐life (Figure 4E), consequently resulting in an amplified fluorescence signal. Intriguingly, the initial detection of the DCNV fluorescence signal was in the liver, but it gradually transitioned toward the lungs. However, the fluorescence signal of DNV was primarily localized in the liver. The fluorescence signal in the lung regions of the DCNV group was found to be significantly higher than other groups at 24 h post‐injection (Figure 4B). Moreover, in mice without LPS instillation, DCNV exhibited a prominent accumulation in the liver rather than the lungs (Figure S6, Supporting Information), which underscores the targeting capability of DCNV toward inflammatory lung tissues. Furthermore, to better quantify the organ distribution of DNase I, DNV, and DCNV, the major organs of the mice were harvested and imaged by IVIS at 24 h post‐injection. The DCNV group displayed the highest fluorescence intensity in the lungs compared to the DNV and DNase I groups (Figure 4C). A 2.67‐fold increased lung‐to‐liver ratio was observed in the DCNV group when compared with the DNV group (Figure 4D). In addition, we extended the observation period of mice post‐injection in IVIS to examine the clearance dynamics of DCNV. As shown in Figure S7A,B, (Supporting Information) a gradual degradation of DCNV within the lung and liver was observed over time, as evidenced by the diminishing fluorescence signals. Notably, immunofluorescent staining revealed the fluorescence emanating from DCNV to be colocalized with macrophages (Figure S7C,D, Supporting Information), indicating its clearance through the mononuclear phagocyte system. These results align with the clearance mechanism of cellular exosome‐liked materials reported in previous research.^[^ ^28^ ^]^

### 2.4 DCNV Degraded Pulmonary NETs and Alleviated Inflammatory Damage in an ALI Mouse Model

**Figure 5:**
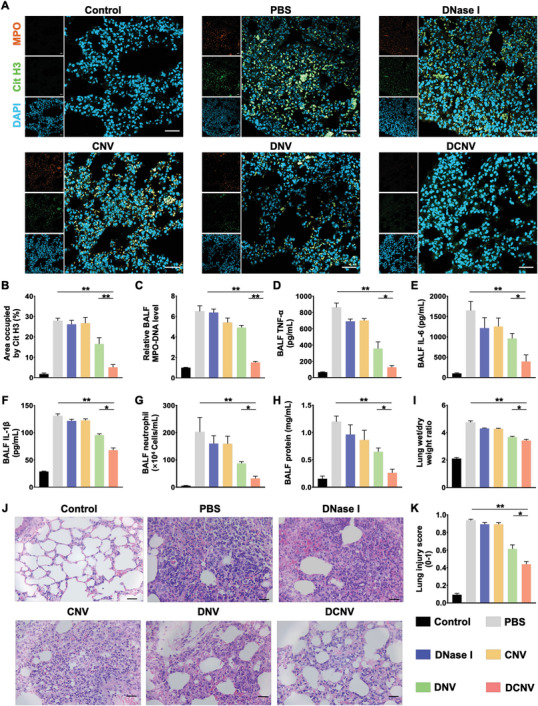
In vivo NETs clearance and therapeutic effect of DCNV in an ALI mouse model. A) Representative CLSM images of NETs immunostaining. CitH3 (green), MPO (orange), and DAPI (blue). Scale bars = 20 µm. B) NETs quantification by the area occupied by CitH3. C) Relative MPO‐DNA levels in BALFs. D–F) levels of pro‐inflammatory cytokines (TNF‐α, IL‐6, and IL‐1β) in BALFs. G) Neutrophil quantification in BALFs. H) Protein quantification in BALFs. I) Lung wet‐to‐dry ratio of indicated groups. J) Representative images of hematoxylin‐eosin‐stained lung slices from indicated groups. Scale bars = 100 µm. K) Lung injury score of indicated groups (n = 5). ^*^p <0.05; ^**^p <0.01.

The therapeutic efficacy of DCNV was evaluated in an LPS‐induced ALI mouse model. The mice were divided into six groups: Control, PBS, DNase I, CNV (cellular nanovesicles expressing CXCR2 without DNase I decoration), DNV, and DCNV. Each group received intravenous injections of the respective formulations (DNase I dose equivalent to 100 U). At 24 h post‐injection, the bronchoalveolar lavage fluids (BALFs) and lung tissues were collected for further analysis. To assess the ability of DCNV to degrade NETs, we visualized extracellular MPO and CitH3 in lung tissue slices using immunofluorescence staining. **Figure** **5**A shows strong colocalized fluorescence between MPO and CitH3 in the PBS, DNase I, and CNV groups, indicating the formation of NETs and negligible clearance after treatment with free DNase I or CNV. However, the level of NETs was significantly reduced in the DCNV group, as revealed by quantification of the CitH3‐stained area, while only a slight decline was observed in the DNV group (Figure 5B). The improved NETs clearance achieved by DCNV could be attributed to the enhanced accumulation of DNase I in the lungs. Moreover, the level of MPO‐DNA complexes in BALFs was also significantly reduced in the DCNV group compared to the other groups (Figure 5C). These results demonstrate the exceptional ability of DCNV to degrade NETs in vivo.

The formation of NETs can exacerbate the inflammatory response in ALI/ARDS by inducing the release of pro‐inflammatory cytokines.^[^ ^29^ ^]^ Therefore, we measured the levels of pro‐inflammatory cytokines including tumor necrosis factor‐α (TNF‐α), interleukin‐1β (IL‐1β), and IL‐6 in BALFs. As depicted in Figure 5D–F, treatment with DCNV resulted in a significant decrease in the levels of TNF‐α, IL‐1β, and IL‐6, in comparison to other groups, indicating that the production of pro‐inflammatory cytokines during ALI can be inhibited by DCNV treatment. Additionally, the infiltration of inflammatory cells, such as neutrophils, was also significantly suppressed (Figure 5G). This reduction in neutrophil infiltration may be attributed to the suppression of the cGAS‐STING pathway, a DNA‐sensing pathway that regulates the expression of inflammatory chemokines and cytokines.^[^ ^30^ ^]^ A recent study has also demonstrated that the inhibition of the cGAS‐STING pathway reduces neutrophil infiltration in the ALI mouse model.^[^ ^31^ ^]^ These results demonstrate that pharmacological inhibition of NETs by DCNV can attenuate the inflammatory response in the ALI mouse model.

As the inflammatory damage progresses, it can cause increased alveolar–capillary permeability, which may lead to the development of pulmonary edema in ALI/ARDS.^[^ ^32^ ^]^ Our results revealed that treatment with DCNV significantly lowered the total protein content in BALFs (Figure 5H), indicating a reduction in alveolar–capillary permeability. Additionally, the lung wet/dry weight ratio was notably reduced in the DCNV group (Figure 5I), suggesting a relief of pulmonary edema by DCNV treatment. Histopathological images of lung tissues also showed that features of lung injury, including alveolar septal thickening, hyaline membrane formation, proteinaceous debris deposition, as well as neutrophil infiltration in the alveolar and interstitial spaces, were reduced after DCNV treatment (Figure 5J). Consequently, DCNV obtained the lowest lung injury score in comparison to the other groups (Figure 5K). Taken together, these results demonstrate the effectiveness of DCNV as a DNase I delivery system that can target inflamed lung tissues to degrade NETs and reduce inflammation damage in an LPS‐induced ALI mouse model. Although further experiments to explore the therapeutic potential of DCNV in other ARDS models are still pending, the capacity of DCNV to effectively clear NETs, as demonstrated in this study, holds great promise for addressing various NETs‐mediated ARDS conditions, such as those involving transfusion and ventilator‐induced lung injury.^[^ ^33^ ^]^

### 2.5 Biosafety Evaluation of DCNV

**Figure 6:**
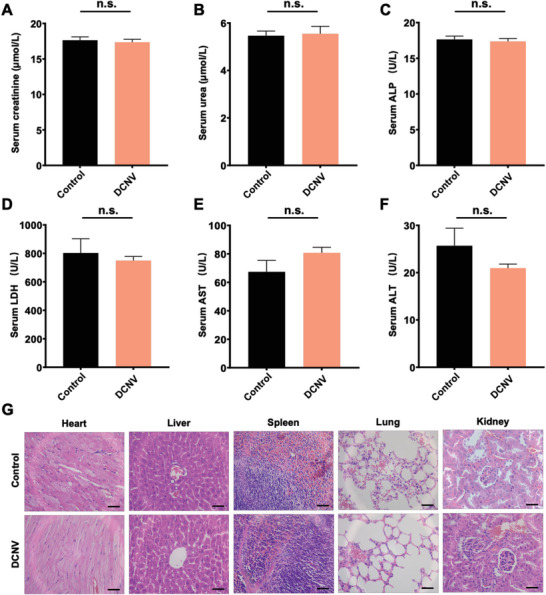
Biosafety evaluation of DCNV. Serum biochemical indexes of mice after DCNV treatment. A) serum creatinine, B) serum urea, C) serum ALP, D) serum LDH, E) serum AST, and F) serum ALT. G) Representative images of hematoxylin‐eosin‐stained organ slices from indicated groups (n = 5). Scale bars = 100 µm. n.s. (not significant) (p> 0.05).

Finally, to evaluate the biosafety of DCNV, a single dose equivalent to 500 U of DNase I, which is five times higher than the treatment dose, was administered to normal C57BL/6 mice. Serum biochemical indices, including creatinine, urea, alkaline phosphatase (ALP), lactate dehydrogenase (LDH), aspartate aminotransferase (AST), and alanine aminotransferase (ALT), were evaluated, and major organs, such as the heart, liver, spleen, lung, and kidney, were subjected to histopathological analysis. The results revealed no significant difference when compared to the normal control group, indicating that DCNV is well‐tolerated even at a much higher dose than the treatment dose and has no apparent toxicity (**Figure** **6**). These findings demonstrate the favorable biosafety profile of DCNV treatment and suggest its great potential for future clinical translation.

## 3 Conclusion

In summary, we reported a novel biomimetic DNase I delivery system, DCNV, that utilized genetically and bioorthogonally engineered cellular nanovesicles. DCNV featured DNase I surface immobilization without compromising its enzymatic activity and exhibited exceptional NETs clearance capacity both in vitro and in vivo. Moreover, by expressing CXCR2 on its surface, DCNV mimicked the inflammatory chemotaxis of neutrophils, leading to enhanced accumulation of DNase I in inflamed lungs after intravenous injection. As a result, DCNV effectively prevented inflammatory damage to lung tissues in an LPS‐induced ALI mouse model. To our best knowledge, this is the first study to use engineered cellular nanovesicles for delivering DNase I in ALI/ARDS. Our findings may provide valuable insights into the development of targeted delivery systems for treating inflammation‐related diseases.

## Conflict of Interest

The authors declare no conflict of interest.

## Supporting information

Supporting Information

## Data Availability

The data that support the findings of this study are available from the corresponding author upon reasonable request.
