# Supplementary material for: Genetically Engineered Cellular Nanovesicle as Targeted DNase I Delivery System for the Clearance of Neutrophil Extracellular Traps in Acute Lung Injury
Source: Adv Sci (Weinh). 2023 Sep 27;10(32):2303053. doi: 10.1002/advs.202303053 (PMC10646266; doi:10.1002/advs.202303053)
Supplement: Supplementary file 1 — Supporting Information [file ADVS-10-2303053-s001.pdf]

ADVANCED SCIENCE
Open Access

Supporting Information

for *Adv. Sci.*, DOI 10.1002/advs.202303053

Genetically Engineered Cellular Nanovesicle as Targeted DNase I Delivery System for the Clearance of Neutrophil Extracellular Traps in Acute Lung Injury

Yang Du, Yining Chen, Fangyuan Li, Zhengwei Mao*, Yuan Ding* and Weilin Wang**

Supporting Information

Genetically Engineered Cellular Nanovesicle as Targeted DNase I Delivery System for the Clearance of Neutrophil Extracellular Traps in Acute Lung Injury

Yang Du, Yining Chen, Fangyuan Li, Zhengwei Mao*, Yuan Ding*, Weilin Wang**

Y. Du[+], Y. Chen[+], F. Li, Z. Mao, Y. Ding, W. Wang
Department of Hepatobiliary and Pancreatic Surgery, the Second Affiliated Hospital, Zhejiang University School of Medicine, Hangzhou, Zhejiang 310009, China
E-mail: wam@zju.edu.cn (W. Wang); dingyuan@zju.edu.cn (Y. Ding); zwmao@zju.edu.cn (Z. Mao); lfy@zju.edu.cn (F. Li)

Y. Du, Y. Chen, F. Li, Z. Mao, Y. Ding, W. Wang
Key Laboratory of Precision Diagnosis and Treatment for Hepatobiliary and Pancreatic Tumor of Zhejiang Province, Hangzhou, Zhejiang 310009, China

Y. Du, Y. Chen, Y. Ding, W. Wang
Research Center of Diagnosis and Treatment Technology for Hepatocellular Carcinoma of Zhejiang Province, Hangzhou, Zhejiang 310009, China

Y. Du, Y. Chen, Y. Ding, W. Wang
National Innovation Center for Fundamental Research on Cancer Medicine, Hangzhou, Zhejiang 310009, China

Y. Du, Y. Chen, Y. Ding, W. Wang
Cancer Center, Zhejiang University, Hangzhou, Zhejiang 310058, China

Y. Du, Y. Chen, Y. Ding, W. Wang
ZJU-Pujian Research & Development Center of Medical Artificial Intelligence for Hepatobiliary and Pancreatic Disease, Hangzhou, Zhejiang 310058, China

Z. Mao
MOE Key Laboratory of Macromolecular Synthesis and Functionalization, Department of Polymer Science and Engineering, Zhejiang University, Hangzhou, Zhejiang 310027, China

F. Li
Institute of Pharmaceutics, Hangzhou Institute of Innovative Medicine, College of Pharmaceutical Sciences, Zhejiang University, Hangzhou, Zhejiang 310058, China

[+] These authors contributed equally to this work.

Experimental Methods

Chemicals and Materials

DBCO-PEG_5-NHS ester was purchased from Confluore (China). N-Azidoacetylmannosamine-tetraacylated (Ac_4ManNAz), Cy5-NHS ester, and Cy5.5-NHS ester were purchased from Aladdin (China). DMEM, RPMI-1640 culture media, Penicillin-Streptomycin (100 ×), and Lipofectamine™ 2000 Transfection Reagent were purchased from Thermo Fisher (USA). PGMLV-CMV-MCS-eGFP-PGK-Puro vehicle plasmid was purchased from Genomeditech (China). Mouse CXCR2 antibody, Human/Mouse Histone H3 (citrulline R2) antibody, and Goat Anti-Rabbit IgG H&L (Alexa Fluor® 488) antibody were purchased from Abcam (UK). ATP1A1 antibody, F4/80 Polyclonal antibody was purchased from Proteintech (China). APC Anti-Mouse CD182 (CXCR2) antibody, Pacific Blue™ Anti-Mouse CD45 antibody, FITC Anti-Mouse/Human CD11b Antibody, and PE-Cy7-Anti-Mouse Ly-6G antibody were purchased from Biolegend (USA). DNase I antibody was purchased from Thermo Fisher (USA). Human/Mouse Myeloperoxidase/MPO antibody and Donkey Anti-Goat IgG NL557-conjugated antibody were purchased from R&D systems (USA). DBCO-Cy5, Histopaque®-1077, Histopaque®-1119, and lipopolysaccharides (LPS) from Escherichia coli O55:B5 were purchased from Sigma-Aldrich (USA). DNase I, bovine serum albumin (BSA), tris buffered saline (TBS), Triton-X-100, and PicoGreen dsDNA quantification assay kit were purchased from Solarbio (China). Phenylmethanesulfonyl fluoride (PMSF), EGTA, Tris-HCl, sucrose, D-mannitol, DAPI, Hoechst 33342, 3,3-dioctadecyloxacarbocyanine perchlorate (DiO), 1,1'-dioctadecyl-3,3,3',3'-tetramethylindocarbocyanine perchlorate (DiI), BCA protein assay kit, and Fluorometric DNase assay kit were purchased from Beyotime (China). The plasmid pBR322 was purchased from Acmec (China). Sytox Green dye was purchased from KeyGEN BioTECH (China). IR-780 iodine was purchased from J&K Scientific (China). Mouse IL-1β ELISA kit, Mouse IL-6 ELISA kit, Mouse TNF-a ELISA kit, and Mouse myeloperoxidase/MPO ELISA kit were purchased from MultiSciences (China). Microsep advance centrifugal devices with omega membrane 3K were purchased from Pall (USA). The μ-slide 4 well was purchased from IBIDI (Germany).

Cell culture

The human embryonic kidney 293T (HEK 293T) cell line was obtained from the National Collection of Authenticated Cell Cultures (Shanghai, China) and cultured following the guidelines provided by ATCC. Cells were incubated in a humidified chamber with 5 % CO_2 at

37 °C. DMEM media containing 10 % of FBS and 1 % penicillin-streptomycin was applied in cell culture.

Construction and characterization of the CXCR2 overexpressed cell line

The gene for murine CXCR2 was cloned into PGMLV-CMV-MCS-eGFP-PGK-Puro lentiviral vehicle plasmid. HEK 293T cells were transfected with the viral packaging vectors psPAX2 and PMD2G plasmid, and lentiviral vehicle plasmid by Lipofectamine™ 2000 Transfection Reagent for 72 h. The lentivirus carrying murine CXCR2 with an eGFP tag in the supernatant was collected via ultracentrifuge (24000 g, 4 °C, 4 h). Then the HEK 293T cells were co-cultured with lentivirus and polybrene (10 μg/mL) for 48 h and received puromycin selection (3 μg/mL) to get the cell line stably expressing CXCR2-eGFP. To confirm the CXCR2 variant on the engineered HEK 293T cell line, pristine and engineered cells were applied for further analysis. The cells were seeded into μ-slide 4 wells at a density of 5×10^5 and stained with DAPI (10 min) and DiI (10 min). Both pristine and engineered cells were monitored under a confocal laser scanning microscopy (CLSM, Zeiss, LSM 900, Germany). To further evaluate the purity of CXCR2 293T, cells were labeled with APC anti-mouse CXCR2 antibody and analyzed by FACSCanto II (BD, USA).

Surface-functionalization of CXCR2 293T cells with azide groups

The CXCR2 293T cells were seeded in μ-slide 4 wells and co-incubated with Ac_4ManNAz (50 μM) for 24 hours. Then the pristine and azido-labeled cells were stained with DBCO-Cy5 (10 μM, 30 min) and DAPI (10 min). Both groups of cells were monitored under the CLSM for confocal imaging.

Generation of CNV-N_3

The CNV-N_3 was prepared according to the references with mild modification.[1] Briefly, the azide-functionalized CXCR2 293T cells were suspended in a hypotonic lysing buffer containing 30 mM Tris-HCl (pH 7.5), 225 mM D-mannitol, 75 mM sucrose, 0.2 mM EGTA, and 1 % PMSF. Then, the cells were disrupted by a Dounce homogenizer with a tight-fitting pestle (20 passes). The homogenized solution received repeated 3 freeze-thaw cycles and was centrifuged at 20000 g for 45 min at 4 °C. The supernatant was then recentrifuged at 100000 g for 40 min at 4°C. The cell membranes were collected and resuspended in PBS. Membrane content was then quantified using a BCA kit. The membranes were sequentially extruded

through 400 nm, 200 nm, and 100 nm polycarbonate membranes (GE Whatman, USA) using a mini-extruder (Avanti, USA) to get the CNV-N_3 vesicles.

Conjugation of DNase I with the DBCO moiety

DNase I was reacted with DBCO-PEG$_5$-NHS ester in PBS (at a molar ratio of 1:3) for 4 h at 4 °C under mild stirring. Unreacted DBCO-PEG$_5$-NHS ester was removed by Centrifugal Filter Device (Pall, USA) at 5000 g at 4 °C for 30 min. DNase I-DBCO was then analyzed by UV-Vis spectroscopy on an Evolution 350 UV-Vis Spectrophotometer (Thermo Fisher, USA).

Preparation and characterization of DCNV

To prepare the DCNV, CNV-N_3 was mixed with DNase I-DBCO in PBS and reacted overnight at 4 °C under gentle rotation. DCNV was collected by centrifuging at 20000 g for 40 min. The quantity of DNase I conjugated on CNV was determined by subtracting the protein content in the supernatant, which was measured using a BCA assay. To visualize the conjugation of CNV-N_3 and DNase I-DBCO, DiO-labeled CNV-N_3 and Cy5-labeled DNase I-DBCO were used to prepare DCNV. The DCNV was then immobilized in a µ-slide 4 well and observed by the CLSM. TEM images were taken on a HITACHI HT7820 (Japan) at a voltage of 80 kV for the characterization of morphology. DLS and zeta potential of DCNV was conducted on a Zetasizer Lab (Malvern, UK). Western blot assay was performed to confirm the presence of CXCR2 and DNase I on DCNV. In brief, DCNV was lysed in RIPA buffer containing 1 % PMSF and heated to 70 °C for 10 min. Samples were then separated by SDS-PAGE and then transferred onto nitrocellulose membranes. The membranes were blocked with 5 % nonfat powdered milk solution for 30 min at room temperature and incubated with primary antibodies overnight at 4 °C. Finally, the membranes were incubated with secondary antibodies and imaged by an Amersham Imager 800 (GE Healthcare, USA).

In vitro DNA degradation and enzymatic activity of DCNV:

To evaluate the DNA degradation capacity of DCNV, 10 µg of pBR322 plasmid DNA was used as substrate in DCNV solution (50 µL) for 10 min at 37 °C, followed by gel electrophoresis as reported by previous work. [2] Moreover, a fluorometric DNase assay was performed to quantify the enzymatic activity of DCNV. The DNase substrate used is a synthetic DNA oligonucleotide probe with a VIC fluorophore (donor) at one end and a BHQ1 quencher (acceptor) at the other end. The absorption spectra of these two fluorescent groups

overlap, and when the distance between them is appropriate, fluorescence energy is transferred from the donor to the acceptor, causing the fluorescence intensity of the donor fluorophore to decrease. When the substrate is cleaved by DNase, the ends of the DNA substrate separate, the two groups move apart, and the fluorescence of VIC is no longer quenched by BHQ1, allowing the fluorescence of VIC to be detected. The enzyme activity of DCNV can be calculated by setting a standard curve using standard DNase I samples. Briefly, 10 μL of 5 × DNase substrates were mixed with 5 μL of 10 × reaction buffer and standard DNase I samples or DCNV. The volume of the solution was adjusted to 50 μL by adding nuclease-free water. The fluorescence intensity emitted by VIC was recorded (Ex = 535 nm/Em = 556 nm) every 30 s for 5 min at 37 °C. Then, the relative fluorescence units (RFU) were plotted against reaction time to calculate the initial velocity of the tested samples. The initial velocities of standard DNase I samples with a range of enzyme activities were also calculated and plotted to create a standard curve. Finally, the relative enzyme activity of DCNV was determined by substituting its initial velocity into the standard curve.

In vitro NETs degradation

Primary mouse neutrophils from the tibia bone marrow were isolated by the previously reported method.[3] Briefly, Histopaque®-1077 and Histopaque®-1119 were used to isolate mouse neutrophils. The purity of neutrophils was ascertained using flow cytometry by calculating the percentage of $CD45^+Ly6G^+CD11b^+$ cells. Then, 1×10^5 isolated cells were resuspended in RMPI 1640 media supplemented with 10 % heat-inactivated fetal bovine serum and seeded in the μ-slide 4 wells. NETs were induced by stimulating the cells with 100 nM PMA. After treatment with PBS, DNase I, or DCNV (at a DNase I dose of 50 U/mL), the cells were stained and observed under CLSM. Extracellular NETs DNA was stained with Sytox Green dye according to the previous work.[4] Meanwhile, CitH3 and MPO were stained for immunofluorescence as previously reported methods.[5] The quantification of the NETs level was determined using Image J software by calculating the percentage of positive SYTOX Green, CitH3, and MPO signals in each field of view.

ALI mouse model

The establishment of an LPS-induced ALI mouse model was carried out following previously published protocols.[6] Briefly, C57BL/6 mice (Gempharmatech, China) at the age of 10-12 weeks were housed in pathogen-free individually ventilated cages with free access to water and chow. An intratracheal instillation of 100 μg LPS in 50 μL of PBS was administered to

the mice.[7] Our investigations were conducted under the guidelines outlined in the Guide for the Care and Use of Laboratory Animals, published by the National Institutes of Health (NIH). The animal experiments were approved by the Institutional Animal Care and Use Committee of Zhejiang University School of Medicine and followed the National Guidelines for Animal Protection (2022-ND-D-131).

In vivo distribution and metabolism of DCNV

The ALI mice after 24 h of LPS instillation were randomly divided into three groups and received intravenous injections of IR780-labeled DNase I, DNV, and DCNV, respectively. The mice were anesthetized and photographed on IVIS Lumina II in vivo imaging system (PerkinElmer, USA) at 0 h, 4 h, 8 h, 12h, and 24h post-injection. For ex vivo fluorescence imaging and tissue distribution analysis, the mice were sacrificed to collect their main organs (heart, liver, spleen, lung, kidney) at 24 h post-injection. To visualize the metabolism of DCNV, Cy 5.5-labeled DCNV was administrated in ALI mice. Major organs of the mice were harvested at indicated time points (24h, 36h, 48h, and 72h post-injection) for IVIS imaging and immunofluorescent staining. The fluorescence intensity was quantified by ROI tools. For immunofluorescence staining, the lower lobes of the right lungs and livers were cut into thin sections (5 μm) and then stained with F4/80 Polyclonal antibody at 4 ℃ overnight. Subsequently, the Goat Anti-Rabbit IgG H&L (Alexa Fluor® 488) antibody was sequentially added and allowed to incubate at room temperature for 1 h. DAPI was applied to stain the nucleus. The slices were then observed under CLSM.

Pharmacokinetics of DCNV in ALI Mice

ALI mice were administered a single intravenous injection of Cy5.5-labeled DNase I or DCNV, both at an equivalent DNase I dosage of 100 U. Plasma samples (30 μL) were collected from the inner canthus veniplex at time intervals of 3, 6, 15, 30, 60, 120, 180, 360, and 720 min post-injection and mixed with 30 μL of heparins at 4 °C.[8] The fluorescence intensity of samples was measured at $\lambda_{ex/em}$ = 670/720 nm. The computation of PK parameters was undertaken using PKSOLVER.[9]

Assessment of pulmonary NETs level

The ALI mice after 24 h of LPS instillation were randomly divided into six groups: Control, PBS, DNase I, CNV, DNV, and DCNV. Each group was intravenously injected with the respective formulations containing a DNase I dose equivalent to 100 U. Bronchoalveolar

lavage fluids (BALF) and lung tissues were collected after 24 h of treatments. Pulmonary NETs level was assessed by measuring the area of CitH3-stained immunofluorescence in lung tissue slices and the level of MPO-DNA complex in BALFs. For immunofluorescence staining, the lower lobes of the right lungs from each group were cut into thin sections (5 μm) and then stained with Mouse MPO antibody and Human/Mouse Histone H3 (citrulline R2) antibody at 4 °C overnight. Subsequently, Donkey Anti-Goat IgG NL557-conjugated antibody and Goat Anti-Rabbit IgG H&L (Alexa Fluor® 488) antibody were sequentially added and allowed to incubate at room temperature for 1 h. DAPI was applied to stain the nucleus. The slices were observed under CLSM and NETs quantification was performed using Image J software by calculating the percentage of positive CitH3 and MPO signals in each field of view. To evaluate the level of MPO-DNA complex in BALFs, an enzyme-linked immunosorbent assay (ELISA) was carried out following the procedure reported in previous studies.[10] Briefly, the BALF samples were diluted and fixed to the pretreated plate of the MPO ELISA kit. After three times washes, the DNA content was measured by the PicoGreen dsDNA quantification assay kit.

Assessment of lung inflammation

Lung inflammation was assessed by measuring the level of pro-inflammatory cytokines and neutrophil counts in BALFs.[11] After treatments, the BALFs were centrifuged at 400 g to collect precipitated cells for leukocyte classification while supernatants were stored for the quantification of pro-inflammatory cytokines. The levels of TNF-α, IL-6, and IL-1β were measured by commercial ELISA kits. The neutrophil counts were analyzed by a BC-5000 vet auto hematology analyzer (Shenzhen Mindray Bio-Medical Electronics Co., Ltd., China).

Assessment of alveolar-capillary damage

Alveolar-capillary damage was evaluated by measuring the protein content in BALFs and the wet/dry weight ratio of lung tissues. A BCA kit was applied to quantify the protein content in BALFs of different groups. In addition, the left lungs were harvested and weighed to measure the wet weights. Then the lungs were dried in an oven for 48 h at 60 °C and weighed to get the dry weights. The ratio of wet weight to dry weight was calculated to reflect the change in lung water content.

Histopathology and lung injury score

After being fixed with 4 % paraformaldehyde for 48 h, the lobes of the right lungs from each group were dehydrated and embedded in paraffin and then cut into 5-µm slices. The slices were mounted on glass slides, dewaxed, and stained with hematoxylin and eosin (H&E). The stained sections were evaluated by pathologists and subjected to standard histopathological analysis according to the Official American Thoracic Society Workshop Report.[12]

Biosafety evaluation

A single dose of DCNV equivalent to 500 U of DNase I was administered to normal C57BL/6 mice. At 7 days after injection, the serum samples were collected and the levels of creatinine, urea, ALP, LDH, AST, and ALT were analyzed by an automated clinical chemistry analyzer (Dirui, China). Moreover, the major organs (heart, liver, spleen, lung, and kidney) were harvested for H&E staining.

Statistical analysis

Data were presented as mean ± SEM. The two-tailed unpaired Student's t-test was applied for statistical analysis among different groups. Differences were considered statistically significant when the p-value was < 0.05. The asterisks denoted the range of p-values: * indicating $p < 0.05$ and ** indicating $p < 0.01$.

Supporting Figures

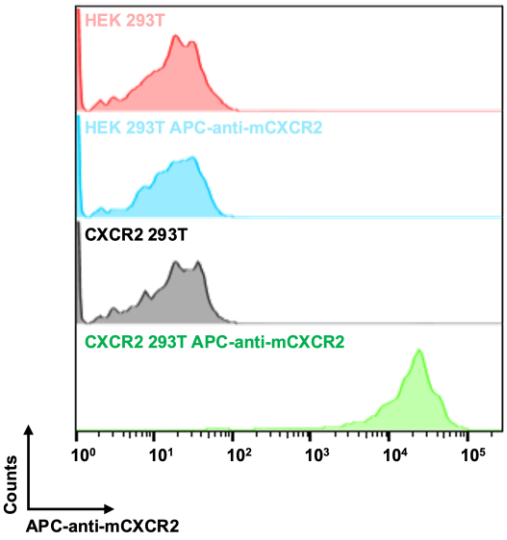

Figure S1. Flow cytometry quantification of CXCR2 expression on HEK 293T cells and CXCR2 293T.

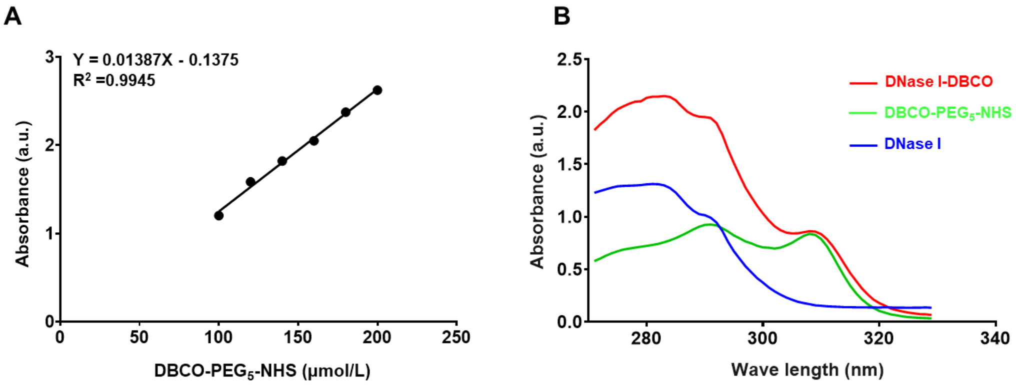

Figure S2. UV-Vis quantification of DNase I-DBCO. (A) The standard curve of DBCO-PEG$_5$-NHS in PBS solution (1% DMSO) at 308 nm. (B) Absorption spectra of DNase I, DBCO-PEG$_5$-NHS, and DNase I-DBCO. The number of DBCO molecules conjugated to DNase I can be calculated by substituting the absorbance of DNase I-DBCO at 308 nm into the standard curve.

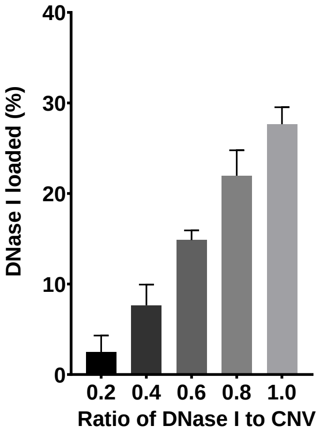

Figure S3. Grafting efficiency of DNase I on the DCNV at different feeding ratios (n=3).

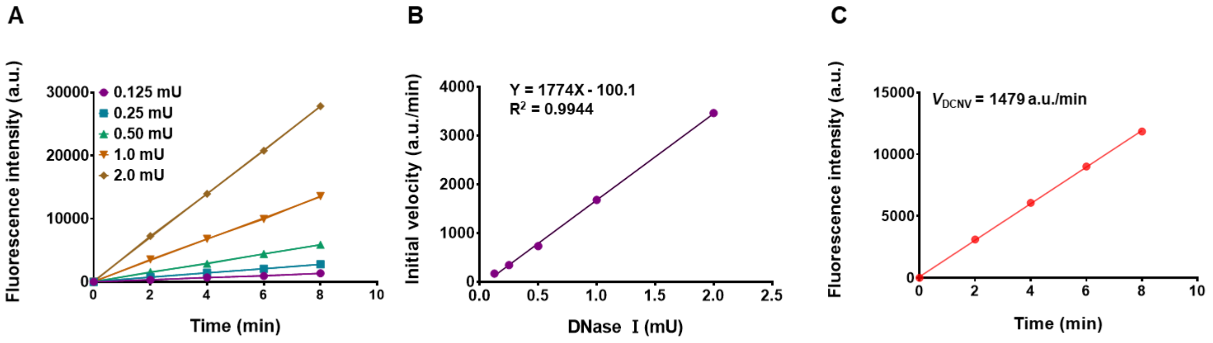

Figure S4. (A) Real-time fluorescence changes of DNA probes incubated with free DNase I of varying enzymatic activity. The slope of the curve represents the initial velocity. (B) Standard curve of DNase I enzymatic activity against initial velocity. (C) Real-time fluorescence changes of DNA probes incubated with DCNV. The enzymatic activity of DCNV can be determined by substituting its initial velocity into the standard curve.

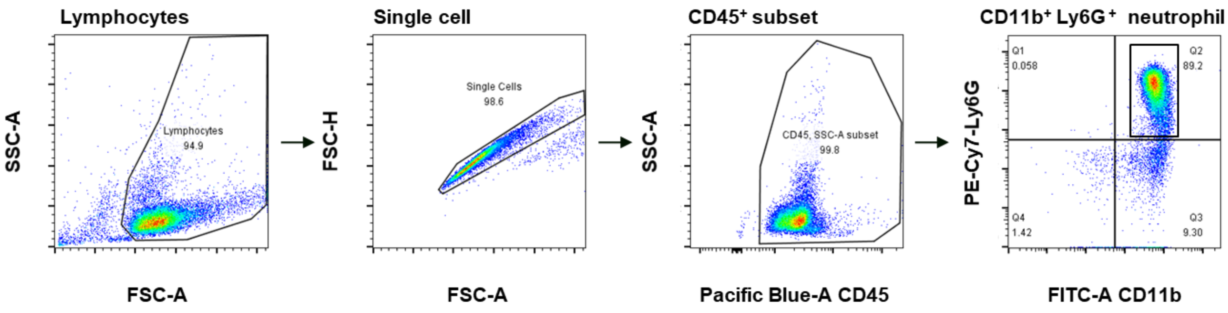

Figure S5. Flow cytometry gating strategies and purity evaluation of isolated neutrophils.

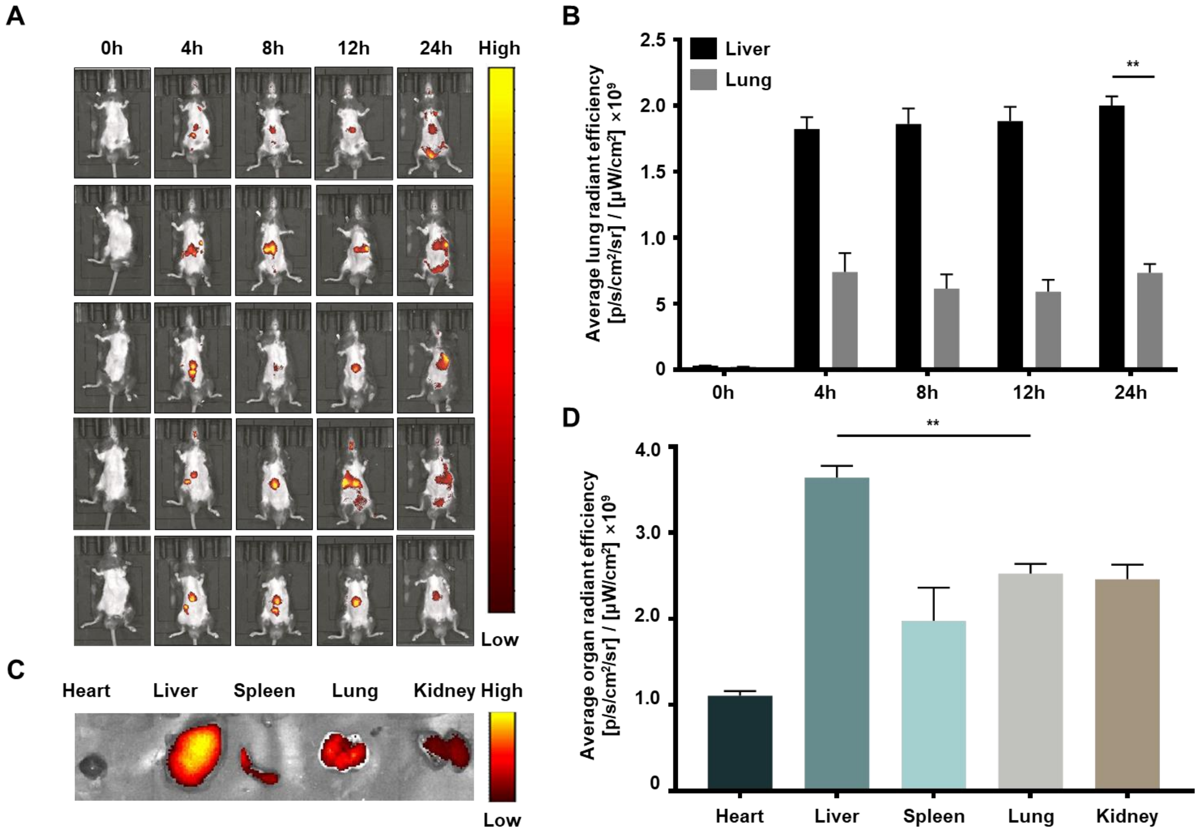

Figure S6. The organ distribution of DCNV in mice without LPS instillation. (A) IVIS images of mice after intravenous injection with IR780-labeled DCNV at indicated time points. (B) Quantitative analysis of the fluorescence intensity in the livers and lungs (n = 5). (C) Representative ex vivo fluorescence image of major organs (heart, liver, spleen, lung, and kidney) harvested at 24 h after DCNV administration. (D) Quantitative analysis of the fluorescence intensity in major organs at 24 h after DCNV administration (n = 5). **, $p < 0.01$.

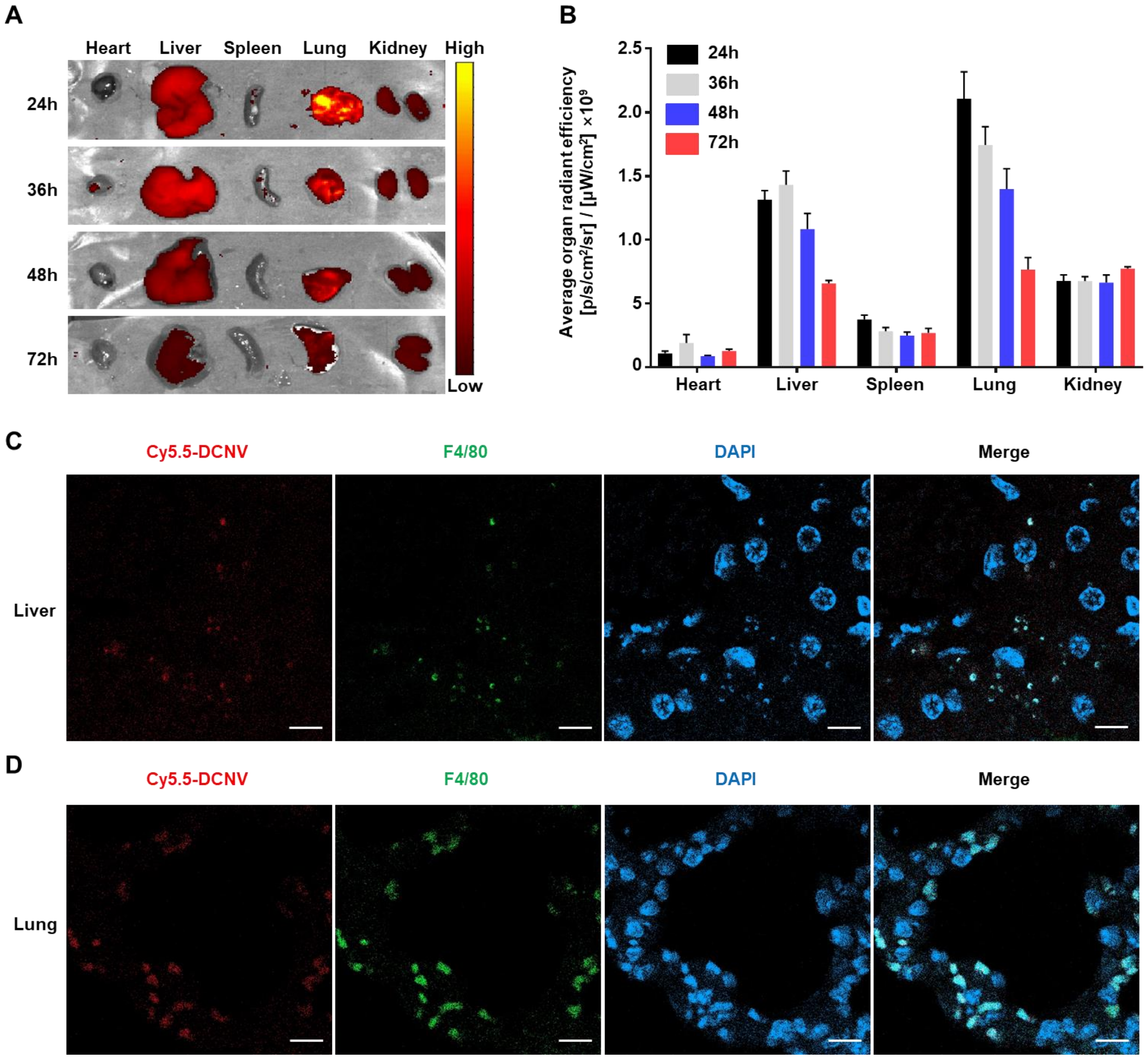

Figure S7. Metabolism and clearance of DCNV. (A) Representative ex vivo fluorescence image of major organs (heart, liver, spleen, lung, and kidney) harvested at indicated time points after DCNV administration. (B) Quantitative analysis of the fluorescence intensity in the major organs (n = 5). (C) Representative CLSM images of Cy5.5-labeled DCNV in the liver. Cy5.5-labeled DCNV (red), DAPI (blue), F4/80 (green). Scale bar = 10 μm. (D) Representative CLSM images of Cy5.5-labeled DCNV in the lung. Cy5.5-labeled DCNV (red), DAPI (blue), F4/80 (green). Scale bar = 10 μm.

References

[1] a) X. Ai, L. Lyu, Y. Zhang, Y. Tang, J. Mu, F. Liu, Y. Zhou, Z. Zuo, G. Liu, B. Xing, *Angew Angew. Chem., Int. Ed.* **2017**, *56*, 3031; b) Q. Zhang, D. Dehaini, Y. Zhang, J. Zhou, X. Chen, L. Zhang, R. H. Fang, W. Gao, L. Zhang, *Nat. Nanotechnol.* **2018**, 13, 1182–1190.

[2] H. H. Park, W. Park, Y. Y. Lee, H. Kim, H. S. Seo, D. W. Choi, H. K. Kwon, D. H. Na, T. H. Kim, Y. B. Choy, J. H. Ahn, W. Lee, C. G. Park, *Adv. Sci.* **2020**, *7* (23), 2001940.

[3] J. Jia, M. Wang, J. Meng, Y. Ma, Y. Wang, N. Miao, J. Teng, D. Zhu, H. Shi, Y. Sun, H. Liu, X. Cheng, Y. Su, J. Ye, H. Chi, T. Liu, Z. Zhou, L. Wan, X. Chen, F. Wang, H. Zhang, J. Ben, J. Wang, C. Yang, Q. Hu, *Nat. Commun.* **2022**, *13* (1), 6804.

[4] T. Kraaij, F. C. Tengstrom, S. W. Kamerling, C. D. Pusey, H. U. Scherer, R. E. Toes, T. J. Rabelink, C. van Kooten, Y. K. Teng, *Autoimmun. Rev.* **2016**, *15* (6), 577-584.

[5] a) L. Yang, Q. Liu, X. Zhang, X. Liu, B. Zhou, J. Chen, D. Huang, J. Li, H. Li, F. Chen, J. Liu, Y. Xing, X. Chen, S. Su, E. Song, *Nature* **2020**, *583* (7814), 133-138; b) K. Yamamoto, H. Yamada, N. Wakana, M. Kikai, K. Terada, N. Wada, S. Motoyama, M. Saburi, T. Sugimoto, D. Kami, T. Ogata, M. Ibi, C. Yabe-Nishimura, S. Matoba, *Biochem. Biophys. Res. Commun.* **2018**, *500* (2), 490-496; c) F. Du, Z. Ding, C. F. Ronnow, M. Rahman, A. Schiopu, H. Thorlacius, *Exp. Cell Res.* **2022**, *421* (2), 113405.

[6] H. Ehrentraut, C. K. Weisheit, S. Frede, T. Hilbert, *J. Visualized Exp.* **2019** (149): e59999.

[7] a) X. L. Huang, X. C. Wei, L. Q. Guo, L. Zhao, X. H. Chen, Y. D. Cui, J. Yuan, D. F. Chen, J. Zhang, *J. Pharmacol. Sci.* **2019**, *140* (3): 228-235; b) J. Wang, R. Huang, Q. Xu, G. Zheng, G. Qiu, M. Ge, Q. Shu, J. Xu, *Crit. Care Med.* **2020**, *48* (7), e599-e610.

[8] D. Huang, Q. Wang, Y. Cao, H. Yang, M. Li, F. Wu, Y. Zhang, G. Chen, Q. Wang, *ACS Nano* **2023**, *17* (5): 5033-5046.

[9] Y. Zhang, M. Huo, J. Zhou, S. Xie, *Comput. Meth. Prog. Bio.* **2010**, *99* (3), 306-314.

[10] a) A. Caudrillier, K. Kessenbrock, B. M. Gilliss, J. X. Nguyen, M. B. Marques, M. Monestier, P. Toy, Z. Werb, M. R. Looney, *J. Clin. Invest.* **2012**, *122* (7), 2661-2671; b) A. J. Dicker, M. L. Crichton, E. G. Pumphrey, A. J. Cassidy, G. Suarez-Cuartin, O. Sibila, E. Furrie, C. J. Fong, W. Ibrahim, G. Brady, G. G. Einarsson, J. S. Elborn, S. Schembri, S. E. Marshall, C. N. A. Palmer, J. D. Chalmers, *J. Allergy Clin. Immunol.* **2018**, *141* (1), 117-127.

[11] Y. Zhou, P. Li, A. J. Goodwin, J. A. Cook, P. V. Halushka, E. Chang, B. Zingarelli, H. Fan, *Crit. Care* **2019**, 23: 1-12.

[12] G. Matute-Bello, G. Downey, B. B. Moore, S. D. Groshong, M. A. Matthay, A. S. Slutsky, W. M. Kuebler, G. Acute Lung Injury in Animals Study, *Am. J. Respir. Cell Mol. Biol.* **2011**, *44* (5), 725-738.
